# Action on commercial gambling—a local government case study from Great Britain

**DOI:** 10.1093/heapro/daag029

**Published:** 2026-08-21

**Authors:** Jenny Blythe, Greg Fell, Anna Twelves

**Affiliations:** School of Medicine and Dentistry, Queen Mary University of London, Whitechapel, London E1 2AD, United Kingdom; London School of Hygiene and Tropical Medicine, Keppel Street, London WC1E 7HT, United Kingdom; Sheffield City Council, Howden House, 1 Union Street, Sheffield S1 2HH, United Kingdom; Sheffield City Council, Howden House, 1 Union Street, Sheffield S1 2HH, United Kingdom

**Keywords:** gambling, local government, premises licensing

## Abstract

Commercial gambling is increasingly recognized as a public health issue, with local government playing a critical role in mitigating its harms. The challenges that public health teams in local government face when there are applications for new gambling premises licences include no mandatory regulatory role; lack of evidence to draw upon and an ‘aim to permit’ principal entrenched in legislation. This case study examines a city council’s refusal to grant a gambling premises licence, and the subsequent legal appeal, which was dismissed in February 2025. Despite the Gambling Act 2005’s ‘aim to permit’ principle, the council denied the application for an Adult Gaming Centre (AGC) due to its proximity to vulnerable populations and sensitive locations. Objections came from public health officials, local residents, and community organizations, citing risks of hidden and invisible gambling harms. The gambling operator appealed, but the court upheld the council’s decision, emphasizing the importance of local knowledge, public health evidence, and the limitations of licencing conditions. The Judge criticized the appellant’s lack of local knowledge and affirmed that authorities are not required to prove direct harm from a yet-to-open venue. The case sets a precedent for integrating public health into licencing decisions and highlights the legitimacy of using ecological data to assess risk. Success was underpinned by a robust policy framework and strategic preparation. The judgement empowers local authorities to prioritize community wellbeing over commercial interests and challenges industry narratives that downplay gambling harms.

Contribution to Health Promotion

- This case study affirmed that public health concerns are legitimate grounds for regulating gambling, even without direct evidence of harm.
- The case study also highlights that the use of ecological and hyper-local data, and localized recognition of hidden harms, are valid strategies, including invisible harms like mental health issues and bereavement.
- The ruling in this case is a template to empower councils to prioritize community wellbeing over commercial interests, setting a precedent for integrating public health into licencing decisions.

## Introduction: gambling harms as a public health concern

Commercial gambling is increasingly recognized as a public health issue, with local government playing a critical role in mitigating its harms (Thomas *et al* 2023). Despite recognition that online gambling increasingly captures a larger percentage of gambling activity, there is evidence from several countries that there is greater availability of what is referred to as ‘land-based’ gambling in areas of deprivation (Alliance for Gambling Reform 2002, Evans and Cross 2021, Pérez *et al*. 2022). In Great Britain, the Gambling Act 2005 mandates Licencing Authorities, situated within local government structures, as responsible for granting premises licences for gambling establishments. These establishments include betting shops, bingo premises, casinos, racing tracks, and adults gaming centres (AGCs). However, the Act’s ‘aim to permit’ principle often conflicts with local knowledge of potential harms, especially in regard to children and other vulnerable communities. This case study focuses on a UK city council’s refusal to grant a gambling premises licence and the subsequent legal appeal, which was dismissed in early 2025, and what has been learnt from the case for future policy and decision makers. Even though it is recognized that licencing laws differ between jurisdictions, there is much to be learnt from this landmark decision for public health practitioners globally.

## Legal framework: the Gambling Act 2005

All local councils in the Great Britain have a gambling policy and statement of licencing principals as mandated by the Gambling Act 2005 (SCC 2024). Under this legislation, licencing authorities must issue premises licences if applications are consistent with the three licencing objectives:

- Preventing gambling from being a source of crime or disorder.
- Ensuring gambling is conducted fairly and openly.
- Protecting children and vulnerable persons from harm or exploitation (Gambling Act 2005).

Despite the ‘aim to permit,’ authorities can refuse applications if they are not ‘reasonably consistent’ with these objectives. In addition, licences can be granted if certain ‘conditions’ are applied (e.g. opening hours stipulations), and objections to premises licence applications can be made by way of ‘representations’. In addition, local public health teams are not what is termed a ‘responsible authority’ for gambling premises licensure, and as such there is no mandatory requirement to inform them of new premises applications, and there is very little evidence to draw upon, from the UK or elsewhere, in terms of population-level, sub-national policies to reduce gambling harms.

## Overview of the city of Sheffield, UK

The city of Sheffield in the UK is the fourth largest in terms of population,with a population of around 560 000 people. However, like many cities in the UK, there are significant health inequalities, and overall Sheffield is more deprived than 70% of other local authority districts in England (Dept for Levelling Up, 2025).

Focusing on gambling, it is estimated that nearly 20 000 adults in Sheffield may benefit from treatment or support for gambling harms or addiction, with thousands more local adults and children in the city experiencing, or at risk of, the harms from someone else’s gambling (OHID 2023).

There are currently 82 licenced land-based gambling premises in Sheffield equating to 14.3 premises per 100 000 population. This is higher than the figure for England as a whole (12.9 premises per 100 000 population) and more than four times that of the local authority area in England with the lowest rate of premises per 100 000 population (3.3 premises per 100 000 population). The majority of Sheffield’s gambling premises are betting shops (60), many of which are located in deprived areas of the city, followed by AGCs (11) (DHSC 2025, Gambling Commission 2025).

Following an inquest into the death of a local resident, which concluded that gambling addiction had led to his suicide, Sheffield City Council has subsequently took action to address gambling harms (Leigh Day 2022). I recently led the development of the city’s first multi-agency gambling harms prevention strategy, with one of the priorities to influence the regulatory environment to prioritize prevention in licencing and planning (SCC 2025).

## The application and refusal

On 6 November 2023, Sheffield City Council’s Licencing Sub-Committee refused an application to open an AGC in the city centre. The decision followed eight formal objections from representations including Green Party councillors, a Local residents’ association, Sheffield Cathedral, and the regional police force alongside Sheffield’s Public Health Service.

The Director of Public Health (author GF) submitted a representation objecting to the application; this was the first time that the Director had objected to a gambling premises licence application. The Director objected on the basis of the risk of preventable gambling harms to children and other vulnerable persons if the Sub-Committee were to approve a licence for the AGC, substantiating this position with detailed evidence.

The Director’s objection included epidemiological evidence on the many harms from gambling and identified population groups most vulnerable to these harms. It also provided information on the estimated costs of gambling harms to the local economy. However, its primary focus was on the location of the proposed AGC and the multiple sensitive locations and areas of risk within the immediate and wider vicinity, which are places frequented by children and vulnerable persons.

In the immediate vicinity, these included a project that provides support to homeless people some of whom have reported experiencing gambling addiction, a premises whose users include addiction support groups, and the Sheffield base of a charity which provides support to refugees and asylum seekers.

Within the wider vicinity, these places included several student residences, university buildings, local parks, and the Sheffield clinic base of the NHS Northern Gambling Service. In total, just over 40 sensitive locations and areas of risk were identified, and a full list and maps of these were submitted with the objection. The objection also noted that there were already eleven existing licenced gambling premises within 500 metres of the proposed AGC and many other places providing commercial gambling products such as pubs (SCC 2023).

The Director of Public Health set out the grounds of the objection at the hearing, drawing from both the epidemiological evidence and the Gambling Commission’s Vulnerability Statement (Gambling Commission 2023). He also spoke of how there are many reasons that someone can be vulnerable to gambling harms, that these may change over time, and that these vulnerabilities may not always be visible making them difficult for gambling operators to identify. The Director also expressed concern that as many as 1 in 12 suicides in England are gambling-related and highlighted the local case of suicide previously mentioned.

This was the first application for a new gambling premises licence in Sheffield for some time and is understood to be the first time that the Licencing Sub-Committee had convened to consider objections to an application. In making its decision to refuse the licence, the Sub-Committee noted the proposed licencing conditions put forward by South Yorkshire Police, however, determined that these would neither be successful nor sufficient in addressing the risks and issues in the area. It balanced the local risks set out in the representations and the statutory ‘aim to permit’, considered the relevant Gambling Commission Guidance and the Council’s Gambling Policy, and concluded that there were no licencing conditions that would sufficiently address the concerns relating to crime or disorder, or the risks to children and other vulnerable persons, in the proposed location.

 

#### The appeal

The gambling operator appealed the decision, leading to a 2-day hearing in late 2024. The Council presented evidence from its Principal Licencing Officer and Director of Public Health (DPH), both asserting that no licencing conditions could mitigate the risks posed by the AGC’s location.

The operators’ witnesses, including the company owner, were criticized for lacking local knowledge and objectivity. The District Judge dismissed the appeal on 24 February 2025, affirming that the Licencing Sub-Committee acted lawfully and appropriately. The judgement emphasized several critical points: firstly, that local knowledge matters (the Judge rejected Royal Amusements’ claim that all city centres are alike, affirming Sheffield’s unique vulnerabilities); secondly, that public health is central (the DPH’s evidence on hidden and invisible harms was pivotal, and the Judge confirmed that public health concerns fall within the Act’s objectives); and thirdly, that licencing conditions are limited(the court found that no conditions could address harms occurring outside the premises or affecting undetectable vulnerable individuals) (SCC 2023).

#### Broader context and industry pushback

The case occurs amid growing concerns about the expansion of land-based gambling. Industry tactics to increase AGC presence in urban areas have prompted resistance from local councils. Sheffield’s stance is part of a broader movement, with local councils in Great Britain campaigning for stronger regulatory powers.

The judgement also challenges industry narratives that downplay gambling harms. It underscores the need for government and regulatory bodies like the Department for Digital, Culture, Media and Sport (DCMS) and the Gambling Commission to prioritize public health over commercial interests.

#### Public health evidence and hidden harms

The testimony from Public Health Services highlighted that gambling harms are complex, often invisible, and not limited to on-premises activity. Vulnerabilities such as mental illness or bereavement are not easily visibly identifiable, making screening ineffective. The judgement acknowledged these challenges, reinforcing the importance of a public health approach. The Council’s evidence-based strategy included emphasizing that the ‘aim to permit’ is not a duty, alignment of its defence with the Council's Gambling Policy Statement of Principles, and was based upon a mutual understanding and shared position between its two witnesses from Licencing Services and Public Health. In addition, the Council drew upon the Gambling Commission’s statement on vulnerability, using hyper-local data on sensitive locations and areas of risk in the proposed AGC location that are frequented by children and other vulnerable persons. Finally, the council approach emphasized the environmental and social risk factors of a new premises, as well as building in direct and indirect lived experience

This approach was validated by the court, which recognized that the ‘aim to permit’ does not override the duty to protect public health.

## Implications for licencing authorities

The case sets a precedent for how local authorities can navigate the tension between the Act’s permissive stance and their duty to protect communities. Local context is crucial: local authorities must use detailed local data to assess risks. Furthermore, Public Health is a legitimate concern: evidence on hidden harms can justify licence refusals. In addition, licencing conditions have limits: local authorities are not obligated to approve licences with conditions if harms cannot be mitigated. Finally, there is judicial support for local decisions: courts may uphold refusals if decisions are evidence-based and aligned with licencing objectives.

## Strategic preparation and policy framework

Sheffield’s success did not happen in isolation. It was underpinned by firstly, a robust gambling policy Statement of Principles which was recently reviewed and updated. This provided the opportunity to information on sensitive locations and areas of risk across the city aligns with the best available evidence on population groups more vulnerable to gambling harms. Secondly, a comprehensive local area profile showed the location of these sensitive areas, and of existing places to gamble, across Sheffield on an online mapping platform. This profile also includes links to sources of local population health data and crime and disorder data. Finally, a proactive gambling harms prevention strategy focused on maximizing the powers, levers, and resources available to Sheffield to prevent and reduce gambling harms, which is underpinned by strong collaborative leadership and political support. These tools enabled the Council to present a compelling, evidence-based case. The judgement affirms that such groundwork is essential for challenging licence applications.

## Legal and evidential nuances

The case also highlights complex issues around evidence and burden of proof. There was no need for direct harm evidence: local authorities are not required to prove harm from a yet-to-open premises. Secondly, licencing decisions are based on likelihood and the balance of probability, and not criminal standards of proof. In addition, Public Health interventions often rely on ecological data rather than individual-level evidence, which courts are increasingly recognizing.

Building on this experience, Public Health practitioners should reflect on the nuances around the bar for burden of proof model and a balance of probabilities approach as they prepare arguments and evidence to present to licencing committees. This shift supports a more holistic, preventative approach to regulation.

## Conclusion: a landmark case for local authority empowerment

The case is a landmark in the regulation of gambling premises in Great Britain. It demonstrates that Licencing Authorities can successfully oppose gambling licences based on public health concerns; courts will support evidence-based decisions that prioritize community wellbeing, and the ‘aim to permit’ principle does not override the duty to protect vulnerable populations. The judgement empowers councils to take a stand against gambling harms and sets a precedent for integrating public health into licencing decisions. It also calls on national regulators to align with this approach and strengthen protections against the proliferation of gambling establishments.

## Data Availability

Not applicable.
